# Supplementary material for: Clinical Cognitive Motor Dissociation: A Case Report Showing How Pitfalls Can Hinder Early Clinical Detection of Awareness
Source: Brain Sci. 2022 Jan 25;12(2):157. doi: 10.3390/brainsci12020157 (PMC8870211; doi:10.3390/brainsci12020157)
Supplement: Supplementary file 1 [file brainsci-12-00157-s001.zip › brainsci-1502930-supplementary.pdf]

Supplementary Table S1. Motor Behavior Tool revised (MBTr)

Item	Notes/instructions	
	Positive signs	
1	*Spontaneous non-reflexive movements*	Observation of the patient without any stimulation. At least one non-reflexive movement defined as non-stereotypical, non-contextualized and non-repetitive intentional motor pattern
2	*Response to command*	Any scorable response to verbal command
3	*Visual fixation or visual pursuit*	Any clearly discernible visual fixation or visual pursuit in any direction
4	*Responses in a motivational context*	Any increase in frequency of non-reflexive motor responses in a salient context (e.g., mother tongue, patient's own name)
5	*Defensive non-reflexive response to a noxious stimulation—Nipple*	Twisting the patient's nipple while keeping the patient's healthier arm between the patient's body and the examiner's arm. Any attempt to push away the examiner's arm that is not a stereotypical posture involving extension and internal rotation of the arms
6	*Defensive non-reflexive response to a noxious stimulation—Nailbed*	Deep pressure to nail beds of four extremities. Any limb movement whose kinematics differs from a motor reflex response in terms of orientation planes and the type of elicited muscles is scored as defensive.
7	*Response to a noxious stimulation—Grimace*	Observation of at least one grimace during administration of noxious stimulation
	Negative signs	
8	Abnormal motor or neurovegetative responses to stimulation	Observation of slow, stereotyped flexion or extension of the upper and/or lower extremities after noxious stimulation or neurovegetative responses (i.e., tachycardia, hypo/hyper-ventilation, hypertension, excessive sweating) to stimulation.
9	Signs of roving eyes or absence of oculocephalic reflex	Slowly roving eyes movements are typical of metabolic encephalopathy indicating diffuse cerebral dysfunction. Oculocephalic responses imply intact brain-stem pathways
